# Shugan Xiaozhi Decoction Attenuates Nonalcoholic Steatohepatitis by Enhancing PPAR**α** and L-FABP Expressions in High-Fat-Fed Rats

**DOI:** 10.1155/2016/7870189

**Published:** 2016-11-28

**Authors:** Yu-feng Xing, Zhen Zhang, Wen-Jun Fu, Da-qiao Zhou, Ailsa Chui-ying Yuen, Daniel Kam-wah Mok, Chi-on Chan, Guang-dong Tong

**Affiliations:** ^1^Department of Hepatology, Shenzhen Traditional Chinese Medicine Hospital, Shenzhen 518033, Hong Kong; ^2^Guangzhou University of Chinese Medicine, Guangzhou 510000, Hong Kong; ^3^State Key Laboratory of Chinese Medicine and Molecular Pharmacology (Incubation), Shenzhen 518057, Hong Kong; ^4^Department of Applied Biology and Chemical Technology, The Hong Kong Polytechnic University, Hung Hom, Kowloon, Hong Kong

## Abstract

This study aimed to investigate the effects of Shugan Xiaozhi decoction (SX) on nonalcoholic steatohepatitis (NASH) induced by high-fat diet in rats. The rats were randomly divided into 6 groups, namely, control, model, fenofibrate, and three different dosage of SX (10, 20, and 40 g/kg/day, p.o.). After establishing the NASH model, at 8 weeks of the experiment, treatments were administrated intragastrically to the fenofibrate and SX groups. All rats were killed after 4 weeks of treatment. Compared with the model group, alanine aminotransferase (ALT), aspartate aminotransferase (AST), free fatty acid (FFA), total cholesterol (TC), triacylglycerol (TG), and low-density lipoprotein cholesterol (LDL) serum in the serum were significantly reduced in all SX treatment groups in a dose-dependent manner. Evidence showed that SX could protect the liver by upregulating the gene and protein expressions of peroxisome proliferator-activated receptor alpha (PPAR*α*) and liver fatty acid binding protein (L-FABP) in a dose-dependent manner. Chemical constituents of SX were further analyzed by ultraperformance liquid chromatography coupled with electrospray ionization mass spectrometry (UPLC-ESI-MS) and 30 chemicals in the ethanolic extract were tentatively identified. To conclude, our results clearly indicated that SX could protect liver functions and relieve hepatic steatosis and inflammation.

## 1. Introduction

The prevalence of nonalcoholic fatty liver disease (NAFLD) is increasing worldwide and the disease is estimated to affect approximately 20% of the population in the developed countries [1]. NAFLD can be further divided into simple steatosis (SS) and nonalcoholic steatohepatitis (NASH) by histological findings [2, 3]. NASH is characterized by the presence of ballooning and lobular inflammation in addition to macrovesicular steatosis [4] and may progress to cirrhosis and hepatocellular carcinoma [5].

The world's overweight population has increased significantly over the past few decades [6]. This is undoubtedly related to changes in lifestyle, particularly in relation to one's diet and the amount of one's physical activity. There is an increase in the number of NASH cases and a decrease in the onset age [7]. Exercising more and weight control are still the major treatment of NASH and drugs for weight loss may help reverse or slow down the progress of the disease. Exercising and weight control do not directly target the liver; they only help to reduce lipid retention through adjusting the metabolism of the body as a whole. Along this line of thought, Traditional Chinese Medicine (TCM), which adopts a holistic approach to regulate and balance various physiological activities of the body, could be an answer to NASH.

In TCM theory, NASH is described in a perspective very different from Western medical terms. The symptoms of NASH could be categorized as hypochondriac “xietong” or liver stuffiness “ganpi.” It is a result of overnutrition in diet (including high-fat diet) or dysfunction of the spleen (our digestive function) in transporting (hence utilizing) the nutrients to other parts of the body, leading to accumulating “dampness” and “heat” in the liver and blocking* qi* circulation in the meridians. The liver* qi* stasis, excessive dampness, and blood stasis in the liver is known as “ganpi.” The TCM treatment of “ganpi” (NASH) focuses on fortifying the spleen, dispelling “phlegm” (dampness accumulated), and soothing the liver qi stasis. Shugan Xiaozhi decoction (SX) was proposed by Xing and Tong [8] with insights gained from two ancient traditional formulae “Sini Tang” (decoction for resuscitation) and “Yinchenhao Tang” (artemisia decoction). The second formula is designed to dispel the phlegm and the first one is designed to fortify* qi* and strengthen the spleen for promoting action to dispel phlegm. Although SX was formulated with a somewhat different description of physiological functions of the human body, our clinical experience suggested that SX is good for NASH patients in the modern sense. After the intake of SX, our results showed that it significantly reduced the blood lipid level and partially restored the functions of liver [9]. More research on this formulation would be needed to understand the actions of SX and to develop a better treatment for NASH patients.

In this study, we aimed to investigate the underlying mechanism of SX in rats with NASH induced by high-fat diet, in order to gather pharmacological data to support its modern clinical uses. Biological parameters and endpoints including alanine aminotransferase (ALT), aspartate aminotransferase (AST), free fatty acid (FFA), total cholesterol (TC), triacylglycerol (TG), and low-density lipoprotein cholesterol (LDL) levels were measured, and some gene and protein expressions from the liver that might be related to the actions of SX were further evaluated. Our results demonstrated for the first time that SX upregulated peroxisome proliferator-activated receptor alpha (PPAR*α*) and liver fatty acid binding protein (L-FABP) in the liver, which contributed to the therapeutic effects of SX in the treatment of NASH. Finally, chemical analysis of SX was performed to provide a better understanding of the chemical constituents and its liver protection effect.

## 2. Materials and Methods

### 2.1. Animals

Male Sprague–Dawley rats weighing about 200 ± 20 g were obtained from Experimental Animal Science Center of Guangzhou University of Chinese Medicine (license number SYXK (Yue) 2013-0001). The use of animals in this study was approved by the Ethics Committee of Guangzhou University of Chinese Medicine. The rats were housed in specific-pathogen-free grade animal facility under standard conditions (temperature of 22 ± 2°C, 12/12 h light/dark cycle, and humidity of 50 ± 10%) with free access to water.

### 2.2. Animal Grouping, Modeling, and Drugs

90 rats were randomly divided into 6 groups of 15: these groups were control group, high-fat diet group (HFD), treatment groups with low dosage of SX (10 g/kg/day, p.o.) (HFD + LSX), medium dosage of SX (20 g/kg/day, p.o.) (HFD + MSX), and high dosage of SX (40 g/kg/day, p.o.) (HFD + HSX), and treatment group with fenofibrate (0.1 g/kg/day, p.o.). At the beginning of the first 8 weeks of the experiment, the control group was fed with a normal diet [composition: protein (14%), fat (10%), and carbohydrate (76%)], while the other groups were all fed with a high-fat diet (HFD) which is a standard rat chow supplement with cholesterol (1%), protein (10%), and lard (10%). All chow was obtained from Guangdong Provincial Medical Laboratory Animal Center (Guangdong, China). Model evaluation was done by point counting, in which 5 rats of each group were selected randomly and their liver tissues were observed under a microscope for confirmation. After the model building stage, all the rats in the treatment groups were ig given fenofibrate or SX extracts while the rats in the normal and high-fat diet groups were fed with the same dose of distilled water. The course of treatment was 4 weeks. At the end of the treatment period, rats were fasted overnight and sacrificed by cervical dislocation. Tissue samples (blood and livers) were collected and stored in freezer at −80°C.

SX is composed of twelve Chinese Materia Medica (CMMs): Bupleuri Radix, Paeoniae radix alba (stir-baked), Aurantii Fructus Immaturus, Glycyrrhizae Radix et Rhizoma, Artemisiae Scopariae Herba, Gardeniae Fructus, Poria, Alismatis Rhizoma, Crataegi Fructus, Cassiae Semen, Nelumbinis Folium, and Pumex in a ratio of 2 : 1 : 3 : 1 : 6 : 2 : 4 : 6 : 6 : 6 : 6 : 6. All the CMMs were in the form of formula granules and purchased from Guangdong Yifang Pharmaceutical Co., Ltd. Fenofibrate capsules were purchased from Laboratoires Fournier S.A. (batch number H20140369).

### 2.3. Reagents

ALT, AST, TG, TC, and LDL-C kits were purchased from Shanghai Kehua Bio-Engineering Co., Ltd; FFA kits and Trizol were purchased from AmyJet Scientific Inc and TAKARA, respectively. qRT-PCR kits and solvent were purchased from DBI Bioscience. Cell lysis buffer and antibodies against PPAR*α*, L-FABP, long chain acyl-CoA dehydrogenases (LCAD), carnitine palmitoyltransferase-1 (CPT-1), acyl-CoA oxidase (ACO), and glyceraldehyde 3-phosphate dehydrogenase (GAPDH) were obtained from Cell Signaling Technology (Beverly, MA, USA).

HPLC grade acetonitrile, formic acid, and analytical grade ethanol were purchased from Tedia (USA). Double deionized water was prepared by a Milli-Q-water-purification system (Millipore, MA, USA). All other chemicals used were of analytical grade.

### 2.4. Liver Histopathological Examination

4% paraformaldehyde-fixed livers were processed and embedded in paraffin according to routine histologic techniques. Liver sections, 5 *µ*m in thickness, were stained with Hematoxylin and Eosin (H&E) and examined under a light microscope. In addition, lipid accumulation was further assessed by Sudan III stain on the cryostat instrument (Leica 1850, Leica Microsystem, Germany).

### 2.5. Biochemical Analysis

ALT, AST, FFA, TC, TG, and LDL-C were determined with an automatic biochemical analyzer (ADVIA 2400 Clinical Chemistry System, Siemens, Germany).

### 2.6. Real-Time Reverse Transcription-Polymerase Chain Reaction (qRT-PCR) Analysis

To determine the gene expression levels, total RNA was isolated from the liver using Trizol reagent according to the standard protocol. 2.0 *µ*g of total RNA was reverse transcribed into cDNA using RevertAid first-strand cDNA synthesis kit.

Total reaction mixture (20 *μ*L) containing 0.2 *μ*M of individual primer set (Table 1) was prepared to determine the mRNA expressions of PPAR*α*, L-FABP, LCAD, CPT-1, ACO, and GAPDH by using real-time RT-PCR procedures [Agilent Stratagene Sequence Mx3000P Detection System with quantitative PCR SuperMix kit (Invitrogen)]. Expression levels of cDNA were expressed as ratios of mRNA expression of individual gene to that of the housekeeping gene GAPDH, in the corresponding samples.

The qRT-PCR reaction procedures are as follows: 94°C, 2 min; 94°C, 20 s; 58°C, 20 s; 72°C, 20 s; 40 cycles. Each qRT-PCR reaction was performed in triplicate.

### 2.7. Western Blot Assay

Briefly, rat livers were homogenized in lysis buffer and centrifuged at 14,000 rpm at 4°C for 10 min. Protein was quantified by the BCA assay (Pierce, Rockford, IL, USA). The protein (20 *μ*g) was resolved on a 10% SDS-polyacrylamide gel and transferred to a PVDF membrane. After blocking the membrane with 5% milk in TBST for 1 h, signals were detected with primary antibodies overnight at 4°C. For protein detection, blots were incubated with horseradish-peroxidase-conjugated secondary antibody for 1 h at room temperature, followed by ECL detection. Densitometric measurements of band intensity in the Western blots were performed using Image J Software.

### 2.8. Chemical Analysis of SX

#### 2.8.1. Extraction Procedure

Around 0.3 g powder sample was placed into a 50 mL centrifuge tube and then sonicated with 10 mL 70% ethanol for 15 minutes at room temperature. Then, the mixture was centrifuged at 14000 rpm in 4°C for 15 minutes. 100 *μ*L supernatant was obtained and added into 900 *µ*L of 70% ethanol. The solution was filtered through 0.45 *µ*m membrane filters prior to chemical analysis.

#### 2.8.2. Ultraperformance Liquid Chromatography Coupled with Electrospray Ionization Mass Spectrometry (UPLC-ESI-MS)

UPLC-ESI-MS analysis was performed on Orbitrap Fusion Lumos Tribrid Mass Spectrometer (ThermoFisher Scientific, USA) with UPLC (ACQUITY UPLC® System, Waters, USA). A UPLC C18 analytical column (2.1 mm × 100 mm, ID 1.8 *µ*m, ACQUITY UPLC HSS, Waters, USA) was used for separation at room temperature of 20°C. The mobile phase was a mixture of water (A) and acetonitrile (B), both containing 0.1% (v/v) formic acid, with a linear gradient elution as follows: 0–2 min, 98% A; 2–30 min, 35% A; 30–35 min, 5% A; 35–40 min, 5% A; 40–40.5 min, 98% A; 40.5–44 min, 98% A. The injection volume was 3 *µ*L. The flow rate was set at 0.30 mL/min.

Heated Electrospray ionization (HESI) was used in both positive and negative ion modes. The operation parameters were as follows: spray voltage static (positive ion 3600 V, negative ion 3000 V) sheath gas flow rate 20 units, aux gas flow rate 10 units, sweep gas 10 units, ion transfer tube temperature 300°C, and vaporizer temperature 200°C. The mass spectra were acquired in full scan mode from 100 to 1200 in mass to charge ratio (*m*/*z*) with a mass resolution of 120000, RF Lens 30%. The mass spectra were collected in centroid mode. Data were evaluated by Quan/Qual Browser and Xcalibur 4.0 (ThermoFisher Scientific, USA).

### 2.9. Statistical Analysis

All data were shown as the mean ± standard deviation (SD), and one-way ANOVA was used to calculate significant differences between the groups. Statistical differences were considered to be significant if* p* was less than 0.05. All statistical analyses were performed using SPSS 19.0 (SPSS Inc., USA.).

## 3. Results

### 3.1. Morphology of Liver Tissue

**Figure 1:**
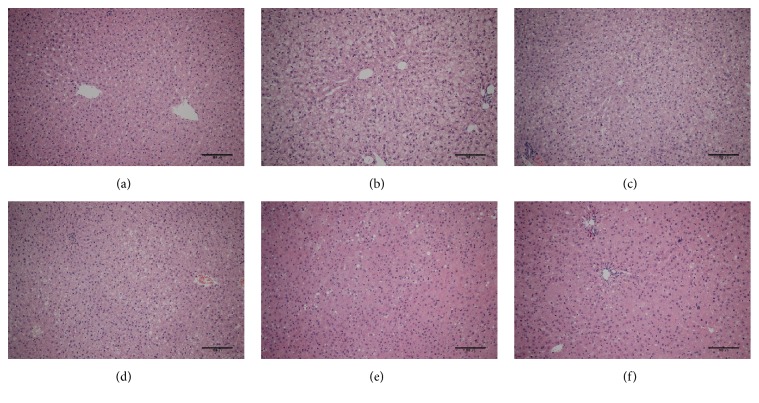
Histological changes of liver section in different groups (H&E staining): (a) control, (b) HFD, (c) treatment of LSX, (d) treatment of MSX, (e) treatment of HSX, and (f) treatment of fenofibrate; bar = 100 *µ*m.

According to the results of H&E staining (Figure 1), the livers of the control (normal) rats illustrated intact cellular structure (Figure 1(a)). In contrast, the livers of rats from the NASH rat model group (Figure 1(b)) revealed mild macrovesicular steatosis, ballooned hepatocytes, and focal intralobular inflammation with extensive lipid depositions. The above pathological changes indicated that the model was successful. In animals given SX and fenofibrate (Figures 1(c)–1(f)), a lesser degree of lipid deposition in hepatocytes was observed and inflammation was improved.

**Figure 2:**
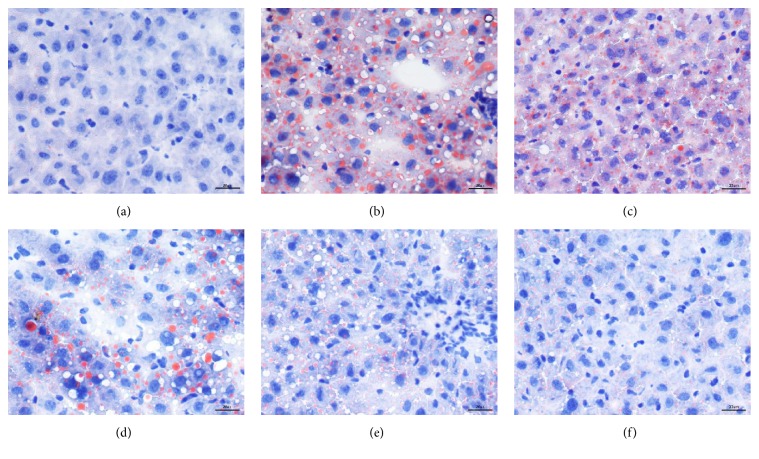
Histological changes of liver section in different groups (Sudan III staining): (a) control, (b) HFD, (c) treatment of LSX, (d) treatment of MSX, (e) treatment of HSX, and (f) treatment of fenofibrate; bar = 20 *µ*m.

Intracellular lipid deposition in hepatocytes was clearly shown via Sudan III staining and the results were illustrated in Figure 2. The livers of HFD rats showed much more droplets (in orange) in the peripheral periportal zone in addition to the central venous zone (Figure 2(b)). In animals given SX and fenofibrate (Figures 2(c)–2(f)), a lesser degree of lipid deposition in hepatocytes was observed, especially in the treatment groups with a high dosage of SX and fenofibrate.

### 3.2. Effect of SX on the Activities of ALT and AST in Serum

The results of the treatment of SX on the activities of ALT and AST were summarized in Table 2. As compared with the normal group, the ALT and AST activity of the serum in the model group significantly increased (*p* < 0.01); compared with model group, the ALT and AST activity of the serum in the treatment group significantly decreased (*p* < 0.01).

### 3.3. Effect of SX on the Level of FFA, TC, TG, and LDL-C in Serum

The results of the treatment of SX on the level of FFA, TC, TG, and LDL-C were summarized in Table 3. Compared with the control group, the FFA, TC, TG, and LDL-C increased with a significant difference (*p* < 0.01). The FFA, TC, TG, and LDL-C in each treatment group were significantly lower than those in the model group (*p* < 0.01).

### 3.4. Effect of SX on the Gene Expressions Level of PPAR*α*, L-FABP, LCAD, CPT-1, and ACO in HFD-Induced NASH Rats

**Figure 3:**
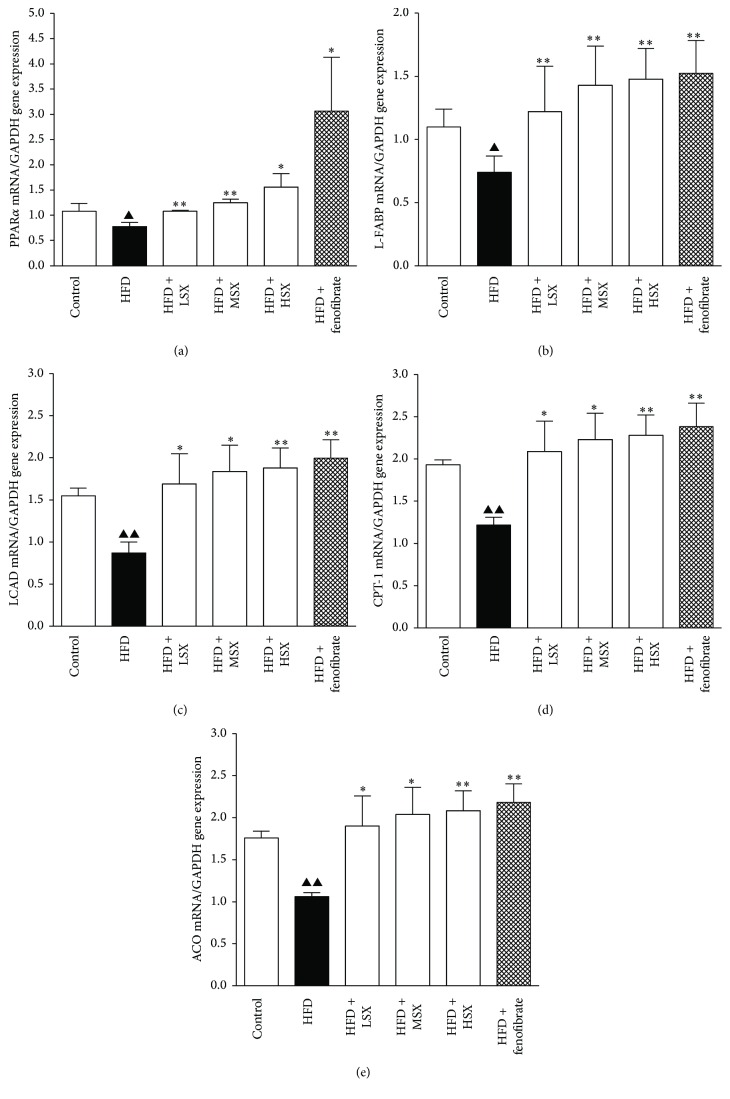
Effects of SX on the gene expressions of (a) PPAR*α*, (b) L-FABP, (c) LCAD, (d) CPT-1, and (e) ACO in NASH rat livers. Data (the ratio of each mRNA to GAPDH mRNA) were expressed as the mean ± SD of three separate experiments. ^▲^ *p*< 0.05 and ^▲▲^ *p*< 0.01 versus normal group; ^*∗*^ *p*< 0.05 and ^*∗∗*^ *p*< 0.01 versus the corresponding HFD-alone group.

The results of qRT-PCR for determining the expression level of various genes in rat livers under different treatments were shown in Figure 3. There was a substantial decrease in PPAR*α*, L-FABP, LCAD, CPT-1, and ACO gene expressions in HFD-induced NASH rats. HFD-induced downregulation of PPAR*α*, L-FABP, LCAD, CPT-1, and ACO mRNA expression could be increased significantly by SX (10–40 g/kg/day) in a concentration-dependent manner (*p* < 0.01). Fenofibrate (0.1 g/kg/day) and positive control group exhibited remarkable upregulation of PPAR*α*, LCAD, CPT-1, ACO, and L-FABP mRNA expressions, compared with the model group.

### 3.5. Effect of SX on the Protein Expressions of PPAR*α* and L-FABP in HFD-Induced NASH Rats

**Figure 4:**
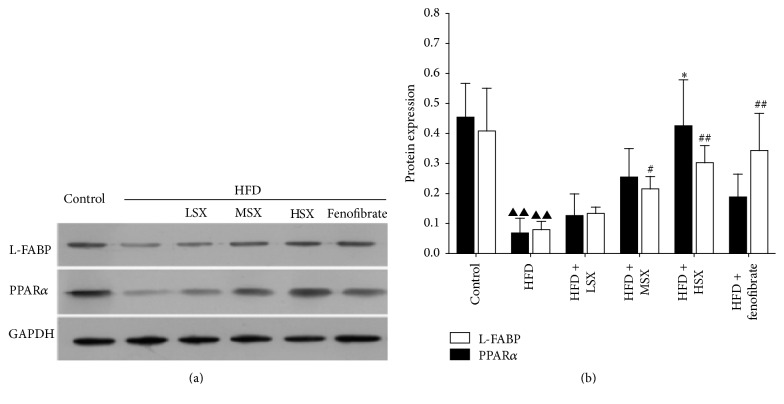
Effects of SX on the protein expressions of PPAR*α* and L-FABP on NASH rat livers. (a) Representative Western blots for PPAR*α* and L-FABP protein expressions on HFD-challenged rats treated with various dosages of SX and fenofibrate. (b) The bands from five independent experiments were quantified by densitometry and represented graphically. Data (the ratio of each protein's expression to GAPDH protein expression) were the mean ± SD of five separate experiments; ^▲▲^ *p*< 0.01 versus normal group; ^*∗*^ *p*< 0.05 versus the HFD group for PPAR*α*; and ^#^ *p*< 0.05 and ^##^ *p*< 0.01 versus the HFD group for L-FABP.

After investigating how SX affected the gene expressions of PPAR*α* and L-FABP, the protein expression levels of PPAR*α* and L-FABP were measured. From the results shown in Figure 4, HFD significantly decreased the PPAR*α* and L-FABP protein level. Interestingly, treating the rats with high dosage of SX (0.426 ± 0.153,* p* < 0.05) could restore the protein expression of PPAR*α* to close to that of the control (0.455 ± 0.112), while SX (10 and 20 g/kg/day) and fenofibrate (0.1 g/kg/day) could not increase PPAR*α* expression under the same experimental conditions. HFD-induced downregulation of L-FABP protein expression could be increased (*p* < 0.05,* p* < 0.01) by SX (20 and 40 g/kg/day) in a concentration-dependent manner. Low dosage of SX (10 g/kg/day) could induce a slight but not significant increase in L-FABP protein expression under HFD-challenged conditions. Additionally, the HFD-mediated downregulation of L-FABP protein level could be upregulated significantly by fenofibrate (0.1 g/kg/day) as compared with the HFD group (*p* < 0.01).

### 3.6. Chemical Profiling of SX

**Figure 5:**
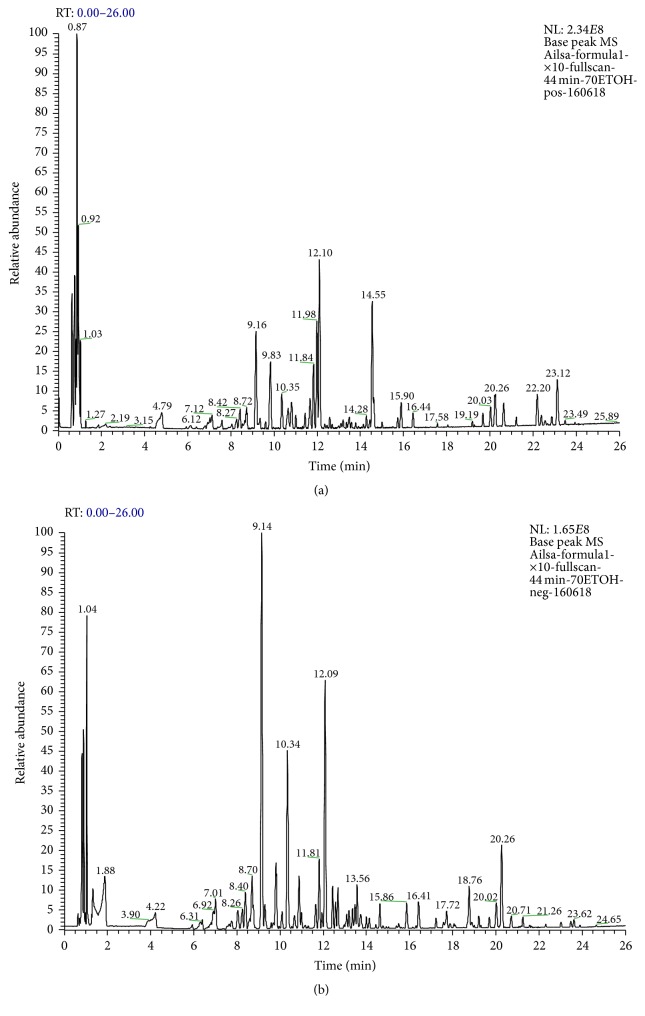
The base peak chromatogram (BPC) of the 70% ethanolic SX extract by UPLC-ESI-MS in the positive (a) and negative (b) ion modes.

In order to provide a better characterization of SX, its chemical profile was determined using UPLC-ESI-MS.  Figure 5 showed the base peak chromatogram (BPC) of the 70% ethanolic SX extract in both positive and negative ion modes. A total of 30 compounds were tentatively identified, by matching empirical molecular, exact mass, and MS/MS fragments with compounds reported in the literatures [10–18], and summarized in Table 4. The mass error for molecular ions of all compounds identified in this study was within ±7 ppm.

## 4. Discussion

NASH is a chronic inflammation of the liver, which is related to environmental factors genetics and metabolisms of the body. The accumulation of lipids in the liver is the major cause of oxidative stress and inflammation leading to hepatic steatosis [19]. The “two hits” theory proposed by Day and James based on their* in vitro* study was widely recognized as the underlying mechanism of the disease development [20]. The hepatic triglyceride accumulation together with low-grade inflammation in the liver initiated the release of the damage-associated molecular patterns (DAMP) molecules from hepatocytes and the pathogen-associated molecular patterns (PAMP) molecules from the gut leading to further damage of liver tissue.

From the “two hits” theory, the increased flux of free fatty acid to the liver in obese and insulin resistance conditions may be important in the development of the disease. The free fatty acids may either go through betaoxidation or esterification to form triglycerides leading to hepatic lipid accumulation. Thus, regulating lipid metabolism could be an important means to manage the disease. Clinically, TC, TG, and LDL-C all increase in NASH patients [21]. Our experimental results are consistent with the clinical observations and there were significant differences in serum FFA, TC, TG, and LDL-C between the HFD model group and the control group. The reduction in serum FFA and lipid levels by SX and fenofibrate certainly played an important role in the therapeutic effects of these agents.

The present animal study clearly showed that SX (low, medium, and high dosages) could reduce the serum ALT, AST, FFA, TC, TG, and LDL-C of rats fed with high-fat diet. In addition, results of histopathological examination of the liver using Sudan III staining indicated that accumulation of fat droplets in the liver was significantly suppressed in the SX treatment groups of all dosages with a dose-dependent trend. This provided strong evidence that SX could regulate lipid metabolism and hence it could protect the liver and prevent the progression of NASH in the rat model. The overall results are consistent with our earlier clinical observation.

The energy expenditure of the body has a close relationship with the oxidation of fatty acids and mitochondrial *β*-oxidation is the dominant oxidative pathway for fatty acids under normal physiological conditions. With regard to fatty acid *β*-oxidation, those key enzymes, including acyl-coenzyme A dehydrogenase (ACADM), CPT-1, and ACO, are transcriptionally regulated by PPAR*α* [22]. In the present work, the gene expression of LCAD, CPT-1, and ACO in the HFD model group decreased significantly compared with that of the control group while the treatments of SX or fenofibrate restored that. In additional, the results showed that LCAD, CPT-1, and ACO had been significantly upregulated in all the SX treatment groups of different dosages with a dose-dependent trend, thereby indicating that SX may protect again NASH by regulating fatty acid *β*-oxidation.

L-FABP and PPAR*α* are two important factors in the pathogenesis of NASH. PPARs are ligand-activated transcription factors that control many pathways in the lipid catabolism. In type 2 diabetes, PPAR agonists are used as lipid-lowering agents and oral hypoglycemic agents [23]. In the liver, PPAR*α* is expressed at high levels in hepatocytes and plays a major role in regulating fatty acid transport and betaoxidation [24]. A protective role for PPAR*α* against liver steatosis and inflammation in NASH has been suggested by the increased susceptibility to NASH of PPAR*α* knockout mice [25, 26]. Liver fatty acid binding protein (L-FABP) is found to promote hepatocyte stress by partitioning potentially lipotoxic long chain fatty acids (LCFAs) into stable triglyceride stores [27]. L-FABP is important in determining the fuel mechanism and promoting the cells to utilize LCFA as a source of energy. Oxidation and esterification of LCFA are markedly reduced in L-FABP knockout mice [28]. Our results showed that the gene expression of PPAR*α* and L-FABP in the HFD model group decreased significantly compared with that of the control group while the treatments of SX or fenofibrate restored that. In addition, PPAR*α* and L-FABP had been significantly upregulated in all the SX treatment groups of different dosages with a dose-dependent trend. This is also consistent with the changes in the protein levels where HFD could downregulate the protein expression of PPAR*α* and L-FABP, while the treatments of SX and fenofibrate both partially restored the protein expression level in a dosage-related manner. Interestingly, treatment with high dosage of SX could restore the protein expression of PPAR*α* (and L-FABP, to a lesser extent) to close to that of the control. All these suggested that modulation of lipid metabolism in the liver is one of the mechanisms for SX to protect the body from the development of NASH. To the best of our knowledge, this is the first study to examine the preventive effects and potential therapeutic benefits of SX on NASH in detail.

There is currently no approved pharmaceutical agent targeting the treatment of NASH. Therefore, developing therapy for the prevention and/or treatment of NASH is an important research area. NASH was not known in Traditional Chinese Medicine theory. The upregulation of L-FABP and PPAR*α* helps the body lower the lipid level and improve the lipid metabolism in the liver. Perhaps this provided a modernized understanding of the ancient Chinese descriptions of dispelling dampness and regulating the liver* qi*.

In the present study, the chemical constituents of SX were further examined by mass spectrometry and multiclass compounds (a total of 30 compounds) including saponins, iridoids and iridoid glycosides, triterpenes and triterpene acids, anthraquinones, flavanone glycosides, flavonoids, and phenolic acids were tentatively identified. Many of these compounds have been reported to possess different biological activities that are related to liver protection. For example, tumulosic acid showed a moderate inhibition effect on nitric oxide (NO) release from lipopolysaccharide- (LPS-) induced RAW 264.7 cells [29]. Saikosaponin A inhibited LPS induced nuclear factor-*κ*B (NF-*κ*B) and interferon regulatory factor 3 (IRF3) activation and the production of tumor necrosis factor-*α* (TNF-*α*), interleukin- (IL-) 1*β*, IL-6 and regulated upon activation normal T-cell expressed and secreted (RANTES) in primary mouse macrophages [30]. Poncirin also inhibited the activation of macrophages stimulated with LPS through the inhibition of LPS binding on Toll-like receptor-4 (TLR4) of macrophages [31]. Paeoniflorin exerted anti-inflammatory effects on primary human hepatic sinusoidal endothelial cells through blocking IL-8 secretion via downregulation of extracellular signal-regulated kinase (ERK) 1/2 and Akt phosphorylation [32]. Naringin prevented carbon tetrachloride-induced acute liver injury in mice by significantly decreasing cytochrome P4502E1 expression, suppressing oxidative stress, inflammation, and apoptosis, and decreasing phosphorylation levels of mitogen-activated protein kinases [33]. Geniposide inhibited alpha-naphthylisothiocyanate-induced hepatotoxicity by downregulating Signal Transducer and Activator of Transcription 3 (STAT3) and NF*κ*B signaling [34]. Thus, based on the chemical profiling of SX obtained, it is suggested that the pharmacological effects of SX may be a synergy of actions of multicomponents. Further studies are needed to elucidate the detailed molecular mechanisms of SX in protecting in the liver.

## 5. Conclusion

Treatment of SX could lower ALT, AST, FFA, TC, TG, and LDL-C in serum and protect HFD-induced liver injury and NASH by upregulation of PPAR*α* and L-FABP. Our experimental evidence supports the use of SX extract as therapeutic agent for clinical NASH treatment.

## Figures and Tables

**Table 1:** The primer sets used for qRT-PCR.

Gene name	Forward primer	Reverse primer
PPAR*α*	5′-ATTTGCCAAGGCTATCCCA-3′	5′-AAGGAGGACAGCATCGTGAAG-3′
L-FABP	5′-TTCTCCGGCAAGTACCAAGT-3′	5′-ATGCACGATTTCTGACACCC-3′
LCAD	5′-AAGAAGTGATCCCTACCACGA-3′	5′-CTCCACCAAAAAGAGGCTAAT-3′
CPT-1	5′-AGCCATGGAGGTTGTCTACG-3′	5′-GGCTTGTCTCAAGTGCTTCC-3′
ACO	5′-CCTTTGTTGTCCCTATCC-3′	5′-GACTCGGCAGGTCATTCA-3′
GAPDH	5′-CCTCGTCTCATAGACAAGATGGT-3′	5′-GGGTAGAGTCATACTGGAACATG-3′

**Table 2:** ALT and AST activities in the different groups of rats.

Group	Dosage [g/kg/day]	ALT (U/L)	AST (U/L)
Control	—	61.25 ± 0.58	101.77 ± 0.69
Model	—	78.99 ± 1.93^▲▲^	130.64 ± 1.40^▲▲^
HFD + LSX	10	69.97 ± 3.91^*∗∗*^	118.30 ± 0.79^*∗∗*^
HFD + MSX	20	67.14 ± 1.37^*∗∗*^	114.93 ± 2.11^*∗∗*^
HFD + HSX	40	64.61 ± 0.73^*∗∗*^	113.30 ± 2.23^*∗∗*^
Fenofibrate	0.1	63.43 ± 0.84^*∗∗*^	112.31 ± 1.46^*∗∗*^

Compared with the normal group, ^▲▲^ *p* < 0.01; compared with the model group, ^*∗∗*^ *p* < 0.01.

**Table 3:** Levels of FFA, TC, TG, and LDL-C in the different groups of rats.

Group	Dosage [g/kg/day]	FFA (nmol/*μ*L)	TC (nmol/L)	TG (nmol/L)	LDL-C (nmol/L)
Control	—	4.16 ± 0.32	3.07 ± 0.20	1.45 ± 0.20	0.93 ± 0.15
*Model*	—	7.77 ± 0.70^▲▲^	5.30 ± 0.62^▲▲^	3.17 ± 0.41^▲▲^	2.80 ± 0.37^▲▲^
HFD + LSX	10	5.49 ± 0.39^*∗∗*^	4.11 ± 0.14^*∗∗*^	2.48 ± 0.40^*∗*^	2.08 ± 0.09^*∗∗*^
HFD + MSX	20	4.88 ± 0.18^*∗∗*^	4.01 ± 0.27^*∗∗*^	2.27 ± 0.43^*∗∗*^	1.83 ± 0.23^*∗∗*^
HFD + HSX	40	4.70 ± 0.40^*∗∗*^	3.97 ± 0.13^*∗∗*^	1.95 ± 0.16^*∗∗*^	1.69 ± 0.20^*∗∗*^
Fenofibrate	0.1	4.55 ± 0.56^*∗∗*^	3.68 ± 0.41^*∗∗*^	1.87 ± 0.27^*∗∗*^	1.48 ± 0.18^*∗∗*^

Compared with the normal group, ^▲▲^ *p* < 0.01; compared with the model group, ^*∗*^ *p* < 0.05 and ^*∗∗*^ *p* < 0.01.

**Table 4:** Compounds identified in SX by UPLC-ESI-MS.

Number	Retention time (min)	Molecular formula	[M − H]^−^ (*m*/*z*) (mass accuracy (ppm))	[M + HCOO]^−^ (*m*/*z*) (mass accuracy (ppm))	[M + H]^+^ (*m*/*z*) (mass accuracy (ppm))	Identification	Origins
1	2.48	C_7_H_6_O_5_	169.0142 (3.55)			Gallic acid	Paeoniae radix alba [10]
2	4.46	C_27_H_32_O_14_	579.1719 (4.83)			Naringin	Aurantii Fructus Immaturus [11]
3	5.86	C_28_H_34_O_15_	609.1825 (4.76)			Neohesperidin	Aurantii Fructus Immaturus [11]
4	6.27	C_28_H_34_O_14_	593.1876 (4.38)			Poncirin	Aurantii Fructus Immaturus [11]
5	6.40	C_17_H_24_O_11_		449.1327 (5.79)		Gardenoside	Gardeniae Fructus [12]
6	8.26	C_23_H_34_O_15_		595.1915 (5.88)		Genipin-1-*β*-gentiobioside	Gardeniae Fructus [12]
7	8.40	C_16_H_18_O_9_	353.0899 (5.95)			Chlorogenic acid	Gardeniae Fructus [12]
8	9.14	C_17_H_24_O_10_		433.1379 (5.77)		Geniposide	Gardeniae Fructus [12]
9	9.82	C_23_H_28_O_11_			481.1676 (−5.82)	Albiflorin	Paeoniae radix alba [10]
10	11.63	C_26_H_30_O_13_	549.1646 (5.18)			Liquiritin-apioside or isomer	Glycyrrhizae Radix et Rhizoma [13]
11	11.81	C_21_H_22_O_9_	417.1216 (5.99)			Liquiritin	Glycyrrhizae Radix et Rhizoma [13]
12	12.43	C_41_H_32_O_26_	939.1170 (6.50)			Pentagalloylglucose	Paeoniae radix alba [10]
13	13.09	C_25_H_24_O_12_	515.1225 (5.82)			Isochlorogenic acid A	Gardeniae Fructus [12]
14	13.47	C_23_H_28_O_11_		525.1644 (5.71)		Paeoniflorin	Paeoniae radix alba [10]
15	15.99	C_32_H_40_O_16_		725.2346 (6.62)		6′′-O-trans-p-cinnamoyl genipingentiobioside	Gardeniae Fructus [12]
16	17.87	C_44_H_64_O_18_			881.412 (−6.24)	22*β*-Acetoxyglycyrrhizin acid	Glycyrrhizae Radix et Rhizoma [13]
17	18.11	C_32_H_44_O_14_		651.2700 (6.45)		cis-Crocin2/trans-crocin2/cis-crocin2′/trans-crocin2′	Gardeniae Fructus [12]
18	19.68	C_30_H_48_O_6_			505.3493 (−6.13)	16-Oxo-alisol A	Alismatis Rhizoma [14]
19	20.25	C_42_H_62_O_16_			823.4068 (−5.22)	Glycyrrhizic acid	Glycyrrhizae Radix et Rhizoma [13]
20	20.26	C_44_H_70_O_14_		867.4799 (5.88)		O-Acetyl-saikosaponin or isomer	Bupleuri Radix [15]
21	20.41	C_32_H_50_O_7_			547.3599 (−5.48)	16-Oxo-alisol A acetate	Alismatis Rhizoma [14]
22	20.76	C_17_H_14_O_7_	329.0685 (5.47)			Aurantio-obtusin	Cassiae Semen [16]
23	20.86	C_42_H_68_O_13_		825.4694 (6.30)		Saikosaponin a	Bupleuri Radix [15]
24	21.08	C_42_H_68_O_13_		825.4693 (6.18)		Saikosaponin d	Bupleuri Radix [15]
25	21.26	C_44_H_70_O_14_		867.4799 (5.88)		O-Acetyl-saikosaponin or isomer	Bupleuri Radix [15]
26	23.47	C_21_H_20_O_11_	367.1187 (4.90)			Kaempferol 3-O-galactoside	Nelumbinis Folium [17]
27	23.52	C_31_H_50_O_4_			487.3391 (−5.54)	Tumulosic acid	Poria [18]
28	23.55	C_30_H_48_O_5_			489.3576 (0.20)	Alisol F	Alismatis Rhizoma [14]
29	24.65	C_16_H_12_O_5_	283.0627 (5.30)			Obtusifolin	Cassiae Semen [16]
30	25.05	C_22_H_22_O_6_			383.1468 (−6.00)	Licoricone	Glycyrrhizae Radix et Rhizoma [13]
